# “Not a Gangster, a Preman!”: Farry Malonda in Indonesia

**DOI:** 10.1007/s10612-025-09850-3

**Published:** 2025-09-12

**Authors:** Laurens Bakker

**Affiliations:** Department of Anthropology, University of Amsterdam, Roeterseiland Campus, Nieuwe Achtergracht 166, 1018 WV Amsterdam, The Netherlands

## Abstract

Can gangsters be good, or can good people use gangster methods? In Indonesia, as in many other countries, the scale between these two is fluid. This article concerns the life history of Farry Malonda, who balanced these two extremes throughout his career. Farry’s past activities as a fulltime preman -a tough guy, a criminal, not adverse to using violence- landed him a reputation that helped him to develop legitimate business activities and social involvement. His activities combine trade, the championing of indigenous rights, nationalism and a public drive for social justice, which has gained him a broad societal position. Whether he acts as a ‘good person’ is important to Farry, and he wants it to be clear that he is not a gangster. Words matter, and this article demonstrates that ambiguous conceptualizations of criminals-cum-security providers may not easily be captured in such a single universal concept.

## Introduction

It is July 2023. As we drive into Sonder, a village in the uplands of the Minahasa region, in the north of the island of Sulawesi in Indonesia, Farry Malonda’s house seems unchanged—albeit a bit dilapidated—since my last pre-Covid 19 visit four years ago. It is a large house with two floors, a wide parking area and a sizeable fishpond to the side. The owner clearly is affluent. Farry opens the fence himself, in bare feet and wearing shorts. We sit on the wide veranda. The house is Farry’s family home, yet he lives there alone. *Bunda* Roosje, his wife of 30 years, passed away a little over a year ago, which impacted him tremendously. Their four children are grown up and live in other parts of Indonesia. Pictures of *Bunda* Roosje, by herself or with Farry, adorn the walls of the veranda. ‘You remember last time you were here, Laurens? You and your wife? Must be four or five years ago. Roosje was still alive. Now it is just you and me, ah, how things change…’.

I first met Farry in 2005. At the time I was researching land conflict in the province of East Kalimantan on the island of Borneo for my PhD thesis. During the research I regularly ran into groups of tough looking men wearing camouflage, army boots and wielding sticks and machetes. These were not the army or the police, but the ‘security wings’ of organizations claiming to represent the indigenous Dayak population and acting to protect their customary rights to land vis-à-vis large-scale commercial plantations and mining companies. While the actions of these groups rarely stopped the actions of such companies, they frequently managed to obtain financial compensation, jobs at the companies or even contracts as local security. The appearance at a company’s office of groups of glowering men (and some women), and the suggestion of their willingness to use violence generally sufficed. I studied these Dayak groups in the context of my PhD research, including extensively interviewing their leaders about their methods and logics (see Bakker, 2010). One of them had told me, ‘you should talk to Farry Malonda. He is no Dayak and does not know about our traditions, but he taught us these strategies’. He then gave me Farry’s mobile phone number.

Meeting Farry took some time. Although he lived in Balikpapan, the business capital of East Kalimantan, at the time, he was away on a prolonged business visit in the Minahasa region. Some three weeks later, however, I was able to arrange to meet him there. I was sitting waiting on the porch of my hotel to be picked up when a black SUV with tinted windows drew up. It sported large ‘Brigade Manguni’ stickers depicting a large owl flying with its claws outstretched. From the car emerged a muscular man dressed completely in black, wearing a cowboy hat, cowboy boots, and a number of gold necklaces. He had large, intricately worked rings with big colourful stones on his fingers and sported a large moustache. He glowered at me from below his hat. ‘Laurens? What do you want? How can I help?’, he asked me, somewhat throwing me with his appearance and directness. I told him about my research and the groups in Kalimantan. ‘Ah yes, them. We taught them all’, he told me, ‘they are strong and brave, but they do not think. They have no experience’. ‘But what do you teach them, then? You are not quite the regular indigenous peoples NGO, right?’, I queried. ‘You do not know about Brigade Manguni?’, he replied, before saying, ‘well, let me tell you, then’. And so began our acquaintance.

In one of our first conversations, I asked Farry, ‘sorry for asking but are you a gangster then?’. He frowned and looked away, seemingly considering the question before launching into an explanation of what it was that he and Brigade Manguni were doing. He clearly took great pride in the organization and seemed to feel that its existence benefitted a greater good. He had been a criminal in the past, he conceded, and his bearing and public appearance clearly echoed that reputation. Yet he also maintained that he was no gangster, nor had he ever been. This confused me, as I felt he fitted that picture quite well, even if he had moved on. Does endorsement—or refusal—of such a denominator by a research subject matter? I think it does, particularly in terms of the societal positioning of one’s role and activities past and present. But if Farry was no gangster, how were we to see him then? Through recounting his life history, this article seeks to answer that question and explore Farry’s—and others—arguments about not being gangsters despite seeming to be gangsters.^1^

What is a gangster? The literature is not conclusive in this respect, suggesting that gangs can be both local ‘teenage peer groups’ that engage in ‘antisocial’ behaviour and ‘street organizations’ involved in criminal activities. Their members ca be part of an ethnic minority, and the gang might exist for a limited time only. Yet gangs can also be regional, or international networks. They can have thousands of members of varied ages and backgrounds, and whereas crime and violence might be central to their image and reputation, they may also be well-established in society and operate quite like social movements (see Brotherton and Barrios 2004; Hazen and Rodgers 2014). Yet words matter and images stick. It matters whether one’s activities are considered legitimate and honourable or labelled criminal offences, both by the state as well as by society. Where governments and judiciaries are weak or corrupt and confusing legal frameworks, wanton law enforcement and poor definitions of property rights allow for—or perhaps necessitate—the emergence of non-state, independent groups as providers of justice and protection (e.g. Jaffe 2012, Goldstein 2005). The question then becomes to what extent does societal benefit mitigate or change the criminal nature of such groups, or whether their security provision merely masks opportunism (cf. Schneider and Schneider 2008: 357–9)?

### A Soldier and a Street Fighter

Farry Malonda was born in the Minahasa region, in the city of Manado, in 1951. When he was fourteen, his parents sent him to a technical school in Semarang, on Java, to study machinery. He found this rather dull, and soon sought an alternative. Tensions with neighbouring Malaysia were increasing at the time, and a prominent army general originating from the Minahasa region had begun to set up a corps of marines. The general wanted a battalion of the corps to be composed of Minahasans, an ethnic group with a strong reputation as tough and loyal soldiers. Hearing this news, Farry reported for the marines, lied about his age, and was accepted as a recruit. In 1967, at the age of sixteen, he was deployed to West Kalimantan. The *konfrontasi* (‘confrontation’) with Malaysia that had predominantly taken place along the Indonesian-Malaysian border had officially just ended. Fighting, however, continued and Farry saw action twice. Most importantly, he felt, he picked up military bearing and stamina. The army made him a tough guy, but once peace broke out army life became dull. In 1971 Farry and others of his battalion took their discharge. Rather than return to the Minahasa region where, as youngsters, they would be subject to the authority of family and traditional societal structures, they opted to seek a more exciting future in the national capital of Jakarta.

The former soldiers could make a living there. Fixed jobs were scarce, but they were taken in by the Minahasan community, who provided them food, places to sleep, as well as *uang rokok* (‘cigarette money’, cash), in exchange for guarding their premises and the neighbourhoods they conglomerated in. Jakarta was a wild place at the time. In 1965–1966 the Indonesian military and police, assisted by armed groups of civilians had killed hundreds of thousands of real and alleged members of the Indonesian Communist Party, ethnic Chinese, non-Muslims, activist farmers and trade union members, thus ‘rescuing’—as the official history put it - the nation from an impending communist coup d’etat. Estimates range from 100,000 to two million victims (e.g. Cribb, 2002:557, Leksana 2021:59). The killings carried two important messages: first, that the danger to Indonesian society came in many shapes and forms, and second, that Indonesians should actively provide security for themselves and those around them in order to guard against such danger. Semi-organized civilian groups had proven their worth in fighting the Dutch for Indonesia’s independence (Cribb, 1991:3–6; Ryter, 2014:153–154), and in defeating the communist threat (see Leksana 2021:61−2). A bunch of tough youths acting as neighbourhood security thus was far from unusual.

The area that Farry and his friends were responsible for ran from the then newly opened department store Sarinah’s—a governmental prestige project that attracted a wealthy clientele and was located at central Jakarta’s prominent Thamrin Street - to the Tanah Abang area, which was—and still is—an economic hub with a huge market, shops, hotels and nightlife. They named themselves ‘Sartana’, taking in these main landmarks.^2^ ‘Yes, we, Sartana, were a gang’, Farry told me. ‘We looked after each other, supported each other like brothers. If somebody had a conflict with a Sartana member, he had a problem with all of us.’ Sartana levied parking fees from those parking vehicles at Sarinah’s, and collected protection money from street sellers, shops and (certain) households. Whereas these sums were generally small, they could add up and the business was lucrative. This would attract raids from neighbouring gangs seeking to take over Sartana’s territory. As Sartana fought off these incursions and expanded their territory, the gang gained a reputation as strong and fearsome fighters. Their clients did not make a fuss about paying fees, and people would come to ask for assistance with problems: conflicts with neighbours, with other gangs, or simply the provision of nighttime security to their premises. As Farry put it, Sartana’s mediation would often suffice to settle the matter peacefully and respectfully.

But Sartana’s members were young, hot-headed and—Farry feels now—quite conceited. They would frequently engage in fights with other gangs - whose members were equally explosive—over territorial infringements, or just ‘for looking at them in a funny way’. Farry recounted an occasion when Sartana members ran into members of another gang in a restaurant. As a result of some vague provocation knives were out in a flash. All the gang members sustained cuts and stabs, the restaurant was in an uproar, and the clientele fled screaming for their lives. ‘That should not have happened’, Farry told me, ‘but it did. It is how we were, then’. And such events happened frequently. Tanamur, Jakarta’s top nightclub in the seventies and long after was located at the edge of the Sartana gang’s territory. A highly profitable location, it was permanently contested. ‘Many fights took place there’, Farry said. ‘There were fist fights, stabbings, shootings… Many Sartana members sustained injuries, but a Minahasan doctor would look after them and stitch them up’.

Sartana had multiple factors going for them. Firstly, they had street credibility. Members looked the part, sporting open shirts, bare biceps, long hair and moustaches. Several of them, including Farry, had ‘Sartana’ tattooed on their lower arms. The letter ‘t’ in Farry’s tattoo is embellished with a Christian cross, while others had the ‘S’ depicted as a dollar sign.^3^ Some sported the number 340, which is the number of the article on murder in the Indonesian penal code. Thinking that these could be trophy tattoos, I asked Farry whether he had actually murdered anyone. ‘No way!’, he replied, laughing, ‘we were just young, trying to look tough’. Secondly, the gang members had a reputation for decency within their own Minahasan community in Jakarta. ‘We were all away from home, in Java, in a foreign city. We needed to stick together, to help’, Farry explained. Without the support of the Minahasan doctor, those offering rooms and accommodation, and of Minahasans in the army and the police who protected them from prosecution, Sartana could not have existed. Sartana needed to contribute to the community to be sustained by them, and its members were aware of this and behaved accordingly. ‘We helped them to develop their businesses, protected their shops and offices’, Farry recounts, ‘without us they would have surely been attacked by local gangs seeking to rob them or chase them out of Jakarta’.

Thirdly, many Sartana members were former soldiers. They had seen military action, and they could call on their military connections when necessary. ‘There were so many instances in Jakarta when I should have been imprisoned’, Farry told me, ‘but we had our friends in the military, and the charges would grow smaller and smaller as the case progressed, and eventually disappear. We were lucky that way, but all successful gangs had such backing. Without it, we could not have existed’. However, this backing did not always work. ‘I was actually imprisoned once’, Farry recounted. ‘I had gone home to Tondano to visit my family, and the local police commander had heard of our activities in Jakarta. He did not like it and put me in prison for a couple of weeks. Without a trial.’ Farry’s Jakartan status thus did not travel that well, and back home he was a young no-gooder who needed to be taught how to behave.

### But Is He a Gangster?

On the porch in Sonder in 2023, I returned to my earlier question from our first meeting in 2005: ‘But weren’t you a gangster, then?’. ‘No’, Farry answered firmly. ‘Gangsters…. Those you have in America, in Italy, mafia… They are criminals, bad people. We have those in Indonesia as well, you know, like Herkules Rosario, John Kei, Anton Medan… I know them all, but we never were active together. I had left Jakarta before they became big and active. They call me *kakak* (older brother).’ He then proceeded to show me some pictures on his phone of himself with these figures, who are three of Indonesia’s most notorious criminals. Each has served long prison sentences, and is publicly associated with gross violence, including multiple murders, as well as with affiliations to powerful governmental and elite figures. Yet over time each has repented, found religion, and become regular guests on television talk-shows, in religious fora and at political gatherings, to talk about their new lives. They are now well-off celebrities, whose reformation has added a layer of respectability over their older reputations. What is it that makes them gangsters and not Farry, however? ‘They were too sadistic, too bloody. And they were very big, always wanted more. They did drugs, prostitution, killed people, you know…’, Farry explained. ‘I’m not a gangster, I’m a *preman*!’, he concluded.

The distinction seemed important, and over the next few days, I asked several dozen people around me in Sonder what they thought made someone a gangster, and what made someone a *preman*. Friends, colleagues, even people who just happened to sit next to me in restaurants, as well as taxi drivers. Hardly an organized research method, I concede, but a way to come to a somewhat informed and substantiated picture that offer some possible differences between the two terms. Most of my interlocutors largely associated gangsters with foreign countries, particularly with the USA and Italy. A gang was understood to connote a well-organized group intent on criminal activities regardless of collateral damage. Gangsters are also well-connected, affluent, socially mobile, and ruthless in their quest for personal gain. They are strategic and capable and make serious money. *Preman*, on the other hand, rarely display these qualities. They were perceived as emanating from the lower strata of society, lacking education and connections, and muddling through by levying parking fees and protection money, small-time criminality. It is exceptional for *preman* to become successful, and if that happens it is not through ruthlessness per se, but rather through societal support and social awareness. *Preman*, many of my interlocuters felt, differ from gangsters by having morals, being socially involved, as well as being somewhat amateurish.^4^ ‘They are like us’, one person said when explaining the difference, ‘they make some money and then they lose it again. They are not calculating, cold, like foreigners. And they are a bit stupid as well’.

While there is not much scholarly literature comparing the two concepts, what exists seems to confirm these ideas to some extent. Sudradjat (2021), for example, uses the concepts relatively interchangeably but emphasizes a *preman’s* low social class and generosity in a short piece titled ‘The land of the *preman*, or the gangsters disguised as philanthropists’. Nugroho (2020) offers a more nuanced comparison that adds the role of ethnicity and the established historical-cultural role of *preman* in Indonesian society, while pointing out the somewhat different and more professionalized focus of organized crime. Farry’s resistance to being called a gangster thus appeared to be linked to a particular perception of criminal behaviour that my interlocutors echoed in various ways. This makes it necessary to further discuss ‘*preman*’.

The term refers, generally speaking, to thugs and to hoodlums associated with street crime and violence. They are the local neighbourhood toughs who extort protection money from the street vendors and bus drivers, but also provide some form of security to the local population (cf. Ryter 1998: 48–51, Bakker 2015: 80–82). They can easily be recognized as they hang around on street corners, markets and anywhere people gather. In the general imaginary they are muscled, macho-looking guys with a certain flamboyance, but in reality they often are scrawny and in dire need of a new shirt. What they lack in physical appearance they make up in loudness and rudeness. A *preman* attitude requires swagger, directness, and the potential for violent eruptions. It is about street credibility.

To be effective, *preman* generally require support from both local society and patrons in the police, armed forces, or government. Sartana enjoyed both. A *preman* should demonstrate that he is not afraid to go his own way and is willing to use violence in achieving his goals, but he must also champion his local community and support his patrons.^5^ A successful, respected and feared *preman* is *jago* (literally a ‘cock’): capable, aggressive, independent, demonstrating his leadership (see Wilson 2012: 121–122). As a *preman* leader explained to me (Bakker 2021: 4–5), a *jago* has morality, uses his brain, and follows his religion conscientiously. He has muscles and a sense of responsibility. He knows how to plan, develop a strategy, and be diplomatic. Yet being a *jago* is also associated with crime. He goes and steals in other communities and is a powerbroker who may put his own interests over those of his community (Schulte Nordholt, 1991). He will work with those in power to maintain their order, their rule, regardless of fairness. A *jago* thus provides order in one capacity but is an agent of disorder in another (Wilson 2015: 181). A *preman* can hence be a street thug, unwashed and underfed or, if he is more successful, he is *jago*, polished, pot-bellied, driving an air-conditioned luxurious car to his next meeting with the city mayor or the chief of police. Connections, reputations and activities matter. A successful *preman* almost by necessity needs to have a criminal past by way of credentials, but he should also show societal involvement and be a known - feared and popular - public figure whose morality is unquestioned.

### To East Kalimantan

In Jakarta, Farry had grown unhappy in Sartana. He did not think there was much future in gang life and he wanted to make more of himself. Whereas he had no problem with the parking money and the protection fees, other activities troubled him. Tanamur was a hotbed for prostitution, the sale of strong alcohol, and the beginnings of the drug trade, and some Sartana members were getting involved in those. Farry decided to leave Sartana and Jakarta and seek out a different life, although this meant giving up on his relations and backing in Jakarta. Together with a few like-minded Sartana members, he decided to migrate to East Kalimantan and start a new life there.

As a soldier, Farry had been posted on the western side of the island of Borneo. The island’s eastern side was renowned for its economic development. It was a place of opportunity with the potential to make a fortune. In 1971 Farry started off working briefly in the logging industry, but moved over to the oil drilling industry in 1972. Oil had been pumped since colonial times, but vast new reserves were being discovered at the time, making it clear that the province possessed enormous quantities of fossil fuel. Drilling for crude oil is technically complex and precise work that involves various specialised skills and can be quite dangerous. Farry had no experience, but he learned on the job, working both on land and drilling platforms at sea. The workers were a rough crowd, as were the specialist foreign employees. Many of these were ex-military themselves, particularly the Americans, and besides gaining a command of English and an impressive number of swear words, Farry also found out how to stand his ground in competing over specific jobs with these men. Farry’s experience as a *preman* helped him to hold his own, but his Minahasan background was an advantage as well given that many Minahasans were employed in the oil business. His status as an army veteran stood him in good stead with the many army-owned or affiliated companies and ensured him jobs as well as accurate information about new economic and political developments in the province.

Rapidly, Farry landed permanent employment with the Indonesian branch of Total, a French oil company. Here he would work for nearly thirty years and build his career step by step, eventually reaching a senior level in the organization. He was an experienced driller and a technician, well aware of the specificities of drilling for oil. Through his many contacts he was also very well informed about opportunities, problems and issues in the industry. A few times, Farry was able to help out friends in other companies in dire need of specific machines or parts by bringing them into contact with others whom he knew had them. The gratifications this brought made him realize that this was a profitable side-business and he set up his own company brokering equipment. Whereas this sat uneasily with his job with Total, he conducted this business at home, in restaurants or in coffee houses. Yet no matter how much money came his way, Farry did not grow rich. He would often help others down on their luck, which established him as a respected member of East Kalimantan society, and a known individual in its oil industry. Then, in 1981, his old friend Japto Soerjosoemarno came to visit.

### Joining Pemuda Pancasila

Japto was a friend of Farry’s from Jakarta who, like Farry, had been involved in the gang life there. While Farry had chosen to leave that business, Japto had continued to build and organize. He was the founder of Pemuda Pancasila (Pancasila Youth), a government-sanctioned youth organization that would become the largest and most powerful such group in all of Indonesia (see Ryter 1998). Japto had come to invite Farry to join and help him set up the organization in East Kalimantan. Pemuda Pancasila (Pancasila Youth) has been called a gang, but it was not just a gang per se (see Ryter, 2014). It was also a registered and legal organization that officially exists to defend *Pancasila*, the official Indonesian state ideology, against such internal threats as religious extremists or communism, and to improve the moral and economic welfare of the marginalized youths who were attracted to it. The organization thus combined a rank-and-file of urban poor and petty criminals with visible and moral adherence to the highest ideals of the nation (Ryter 1998:47). The result was a disciplined group that was willing to act, violently, if necessary, for such goals as its leadership indicated. Clad in distinctive brown-orange camouflage uniforms, its members were highly visible and immediately recognizable on the street. Pemuda Pancasila offices, cars, banners and corrugated iron fences were all painted in the same striking pattern, making the organization’s presence a fixture in the street. Pemuda Pancasila members championed the Indonesian government’s values of development and security and as such could be called upon to clear protesters or inhabitants from designated building sites or mining locations, break up strikes or intimidate political opponents. Like Jakarta’s gangs, the organization also provided security for many businesses, who either requested this service or were submitted to it as a way of extortion. Its alliance with the economic elite and official legal status normalized Pemuda Pancasila’s practices which consequently became by and large seen as legitimate (see Ryter, 1998:48–53; Wilson, 2006:266).

Farry accepted Japto’s invitation to join, allowing Farry to establish new connections to the political, army and police authorities in East Kalimantan, as well as revamp some of his old army contacts in Jakarta. The regime was also taking a decidedly less tolerant view towards what it now called ‘wild’ (‘*liar*’) groups—within which it included Sartana—and in early 1983 began an operation to clear the streets in many of the nation’s larger cities of such groups. ‘Mysterious gunmen’ - ‘*penembak misterius*’ in Indonesian led to was referred to as the ‘Petrus’ campaign - wearing balaclavas they would abduct real or suspected petty criminals and gangsters from their homes, torture them, and then shoot or stab them, leaving mutilated corpses in public places (see Barker 1998: 37–41).^6^ Many of these mysterious shooters were army soldiers or police personnel, and the campaign was part of a larger government strategy to let Indonesian society know that the government could control criminals and the population at large (see Siegel 1998: 110). In other words, urban gangs such as Sartana were being brought to heel, with between 5,000 and 10,000 street gangsters extrajudicially killed between 1983 and 1985. Many gangs disappeared as a result, their remaining members often choosing to join the ranks of official groups such as Pemuda Pancasila.

Pemuda Pancasila was established nationwide according to a military-like command and operational structure. In East Kalimantan, the organization helped members obtain jobs in the logging, plantation, mining and oil industries, so entering all the major fields of economic importance. Farry began to become publicly known as Pemuda Pancasila’s spokesperson. It was not uncommon for villagers to protest when companies were about to take over their farming lands, and a common reaction by both government and companies would be to appeal to Pemuda Pancasila for assistance. Farry explained how generally he would appeal to villagers’ common sense to convince them that they would lose in a confrontation with the authorities whereas through negotiation they might be able to obtain a settlement. Sometimes this would work, but not always, and there were times when Pemuda Pancasila members - rather than the police or the army - burned down villages and forcibly removed the inhabitants, yet Farry considered such occasions as failures rather than successes. He much preferred to seek a solution that took the interests of all sides into account and made sure that the villagers felt seen and respected. Yet ‘sometimes there were hot-headed *provokator* among them, who influenced people and made them not open to reason and insisting on conflict’. This saddened him, as he was quite certain that those villagers had no idea what would be coming to them next.

Total, his official employee, was tolerant of his activities in Pemuda Pancasila. They gave him time off whenever these required his attention and would accept that he could be away for days at a time. Farry also became involved in tensions between villagers and Total. In such cases Farry used Pemuda Pancasila as a vehicle to step in and between the parties. ‘I worked for Total, but I was not Total’, he said, recounting how ‘this was an important difference that people needed to be aware of and that I needed to demonstrate’. In this way, Farry was able to build a career in the employment of Total while simultaneously leading Pemuda Pancasila locally. He was aware that not all of Pemuda Pancasila’s activities were fully legal and he readily agreed that not all of the land clearings were fair, but he claimed that he always sought to manage things as best he could. As a result, he gained a respected reputation in the province and would be invited to official events, dinners and parties by government officials, army and police commanders, as well as by members of the local business elite, but he also had friends among the customary leaders of indigenous groups. He was doing well and providing a good life for himself and his family, when in 1998 the long-standing New Order regime of President Soeharto fell.

### Brigade Manguni Indonesia

President Soeharto abdicated following a series of economic, political and social crises that shook Indonesian society. After some 30 years of rule by his New Order government, the country now faced a leadership vacuum. Mass public discontent, including riots and public violence, broke out all over the country, which the leaderless and corrupt government bureaucracy was unable to cope with. Pemuda Pancasila was strongly impacted by this turmoil. Associated with the New Order regime and the Jakartan elite, it suffered a strong setback in support throughout the nation. While in East Kalimantan it was one of a few areas where it actually remained a broad and popular organization. When I asked Farry why he thought this was, he smiled and said that he thought that leaders there did a good job in balancing the various interests in society.

Internationally, observers seriously considered the possibility that Indonesia would disintegrate into a number of smaller countries, possibly after a civil war. That this did not happen was largely because of a clever and daring policy of democratisation and regional autonomy implemented by new president Yusuf Habibie, but various regional and bloody conflicts did erupt. While local, the impact and scale of these conflicts was considerable and disastrous for those impacted. The national army and police were incapable of putting down regional violence or in some cases - for example, in the Moluccas, a group of islands not far from the Minahasa region - became involved on opposing sides. This left the development and settlement of the conflicts largely to local participants. Meanwhile the new national government introduced a decentralization of substantial administrative and fiscal authority to the regional level of government, thus allowing a much large role for regional interests in lower levels of government. Such increased space for the local allowed for prominent and violent regional actors to secure strong positions in regional politics.

In 1999, fighting broke out between Christians and Muslims in the Moluccas. As the Muslim side began receiving support from the mainly Javanese Laskar Jihad militia, Christian refugees started to arrive in the Minahasa region, which is a predominantly Christian area. They brought with them rumours that Laskar Jihad fighters were preparing to attack Manado, the main Minahasan city. Laskar Jihad infiltrators had already arrived, the rumours went, and were trying to mobilize the Muslim minority to take up arms (see Duncan 2005: 32–35). Regional government set up dialogue sessions between the various religious groups, but local citizens also began to organize militias in order to resist the expected invasion. Among these was a group called Brigade Manguni, who took their name from a species of owl endemic to the region, also widely used as a graphic symbol. The main leader was Decky Maengkom, a former Sartana member who had returned to the Minahasa region from Jakarta in the nineties, and who had built a career as an entrepreneur and a construction company. Maengkom established Brigade Manguni in collaboration with the regional government, the church and local *preman*, resulting in a large, well-connected organization with a clear command structure and chapters throughout the area. Many volunteered to join, but Brigade Manguni made a special point of recruiting local *preman* into the organization in order to enlist their prowess and fighting capabilities, selecting them to receive special training from the local military so as to function as irregular auxiliaries to the army and police.

Partly as a result of the existence of organisations such as Brigade Manguni, Minahasan Muslims refused to support Laskar Jihad, who instead turned their attention to the Poso region in Central Sulawesi, where violence had erupted between Muslims and Christians. Brigade Manguni sent fighters to Poso as well, where they operated under the name of *Kelalawar Hitam* (‘black bats’) because they dressed in black and allegedly could fly noiselessly and magically in the dark. As the Indonesian national forces began to get a grip on the conflict, both Laskar Jihad and the Kelalawar Hitam left the region. Returning Kelalawar Hitam members added considerably to the prestige of Brigade Manguni, as they had proven their capabilities and shown great bravery. Brigade Manguni therefore decided to remain as a permanent civilian organization seeking to provide security and other services to Minahasan society. As Pemuda Pancasila had done previously, Brigade Manguni offered various forms of security services and also acted as an intermediary between entrepreneurs, businesses, government and the local population. The organization would help with the recruitment of workers and several of its leaders ran construction companies. It could also block the entry of outside companies into the Minahasa, at least until the latter had agreed to collaborate (see Bakker 2016: 126–128).

Farry supported Brigade Manguni from the beginning. He provided financial assistance and send a shipment of uniforms - black trousers, shirts with the Bridge Manguni logo, army boots—from East Kalimantan. Visiting Brigade Manguni himself, Farry was asked by Decky to help increase the organization’s size and reach by mobilize more people to join its ranks beyond the Minahasa region. Farry agreed, and set up a Brigade Manguni chapter in East Kalimantan and become its leader there.

Brigade Manguni was one of many such organizations that came into being all over Indonesia at this time of democratic transition. Such civil, militant groups were organized, disciplined and uniformed, generally represented specific religious or ethnic groups, or were affiliated to political parties as their dedicated security forces. They fell under the broad category of what in Indonesia is termed ‘*organisasi kemasyarakatan*’ (‘societal organizations’, or *ormas* for short). Most *ormas* state their goals as specifically involving the protecting the interests of the groups they represent, while simultaneously striving for a peaceful and united Indonesia. They generally proclaim Pancasila and the unity of the Indonesian state to govern their activities and carefully frame themselves and their activities within the ideological order of the state, the furthering of its development and the maintenance of its security (see Bakker and Karim 2022). Yet mostly these *ormas* maintain the double-faced societal role and attitude shown in the eighties and nineties by Pemuda Pancasila, and much of the authority of their leaders continues to derive from their being *jago*.

In 2008, with the oil industry slowing down and Farry’s business brokering becoming a side activity to his engagement in Brigade Manguni, he decided to retire after he had set up a chapter of Brigade Manguni in East Kalimantan with himself as its leader. As Minahasans, Brigade Manguni presented itself as a neutral force within local society: they were not part of the local indigenous population nor tied to any of the larger migrant groups in the province, but mainly existed to help protect the peace in society. Peace was somewhat strained, however. Plantation and mining industries had been gaining in economic importance in the 1990s and 2000s, and were laying claim to ever larger areas of land to which villagers and indigenous groups frequently also claimed rights. Often, the latter were supported by *ormas* such as the Dayak group that introduced me to Farry. This led to tensions, conflict and occasionally violence with Farry and Brigade Manguni often asked to step in and restore calm by brokering a deal between the parties.

### Becoming a Tokoh Masyarakat

In August 2017, Farry agreed to meet me in Minahasan restaurant Bunaken Indah in Balikpapan. He is already there before I enter, waiting for me with his huge black car parked in front of the restaurant, sporting large Brigade Manguni Indonesia logos. I find him at the center of a cluster of people around a table at the back. He is dressed in a black shirt, black hat, camouflage pants and wearing a number of ethnic Dayak bracelets and necklaces. Farry introduces me as his friend from the University of Amsterdam who researches Minahasan culture. People nod politely, and the conservation resumes. As I eat and listen, it becomes clear that they are there to discuss a conflict about a piece of land that belongs to one of them but is included in a new mining concession. They ask Farry for help in talking to the company and he suggests involving a local Dayak *ormas* as they provide security for that company. He says he will seek out his contacts and see what can be done. ‘People often come to me if they have such problems’, Farry tells me later. ‘I know people in the company and I know their security. Recently I helped Dayak villagers who wanted to demonstrate against a plantation company. I know the chief of police. Because we go through him we can have the demonstration’.

Farry’s Facebook page illustrates his connections with a steady stream of messages and photographs of him with senior police and army officials, politicians, and individuals from within the Dayak and Minahasan communities. If nothing is happening for a few days he posts pictures from days gone by, with Japto and other senior Pemuda Pancasila leaders, with members of Sartana or with other illustrious figures whom he has met in the past. Farry is a ‘*tokoh masyarakat*’, a public figure and while many people in Balikpapan do not know him by name they know him by appearance. His Facebook page ensures that he is never far away on the timelines of his many followers and friends. A *tokoh masyarakat* like Farry can similarly mobilize contacts from various backgrounds to provide for whatever needs arranging. If the police and the judiciary are unlikely to solve a citizen’s problem if this involves a powerful or wealthy opponent, going through a channel such as Farry provides an alternative. Parties generally will pay attention as they too are aware of Farry’s reputation and contacts. They know that this is an effective and socially acceptable channel through which to reach a settlement and that there might be consequences if they do not.

At the same time, Farry and his wife *Bunda* Roosje also increasingly returned to their home in Sonder, in the Minahasa region. They enjoyed spending time with their extended families and friends, basking in Farry’s reputation as a successful businessman who had made his fortune in Kalimantan. For some years they raised pigs in Sonder as a new source of revenue, but these died from a disease. Farry regularly returned to Balikpapan when he was called upon by contacts there, bur his and *Bunda* Roosje’s intention was to sell up in Balikpapan and enjoy life together in the Minahasa region. Roosje’s passing put an end to that plan, and although Farry continued to move between the Minahasa region and Kalimantan, he increasingly spent more time in the latter, again stepping up his business interests there.

## Conclusion

On the porch in Sonder in 2023, I reconsider my original question: Should Farry be seen as a gangster? Although, it’s not a label he likes. Farry accepts that he can be considered a *preman*, however, because to him – and others - this indicates bravado and courage as well as societal involvement. But when I push him on the question, he eventually tells me that he prefers to be considered a *tokoh masyarakat*, the more respectable and perhaps more sedate ‘community leader’. He is no longer the street fighter that he was in his youth in Jakarta, even though he likes to keep that memory alive in peoples’ perception of him, making sure that it is known that he can call on the muscle of Pemuda Pancasila or Brigade Manguni if he needs it. But he rarely does, preferring to use the smiles and handshakes of the government officials and societal leaders he knows and with whom he associates. ‘But aren’t you still in a gang?’ I ask him teasingly, ‘just no longer with street *preman*, but with politicians, entrepreneurs, police and army commanders instead?’ Farry does not laugh or smile, disliking the violent image of gangsters, their rudeness, and their dedication to crime. ‘Gangsters deal drugs, force women into prostitution, they do bad things, they have no morals’, he tells me again. ‘I do not want to be involved in such business… I have a conscience. God sees us, you know.’

Ultimately, the question whether Farry is a gangster or not is perhaps not the relevant one to be asking. It can be argued that it provides the wrong lens to understand his life and career. Sartana, in Jakarta, might tick many boxes associated with the concept of a gang as encountered elsewhere, but Pemuda Pancasila moves beyond this, and Brigade Manguni is a further step away given its emphasis on societal security and collaboration with the state. If Farry was once a gangster, he has moved beyond crime. Yet neither Pemuda Pancasila nor Brigade Manguni constitute full departures from illicit and illegal behaviors. The link remains important. If Farry were a gangster in Sartana, he left that lifestyle by getting a respectable job in the oil industry, although establishing himself within this industry owed much to his gangster past. He further built his parallel Pemuda Pancasila and Brigade Manguni careers on the same basis. Although the objective was different. A ‘gangster’ today, in Farry’s perception is a cold-hearted, egoistic individual lacking in morality. While a *preman* is not perfect and may be violent, coarse and criminal, he has morals and his role in Indonesian society is widely known, understood, and often admired. Seen from this perspective, if *preman* are gangsters, they are a particular Indonesian form of the phenomenon, highlighting its fundamental ambiguity and multiplicity.

.

## Footnotes

1 It is important to note that ‘Farry Malonda’ is not a pseudonym. Farry refuses to use a pseudonym and would not agree to the publication of this article unless his real name was used.

2 A brief history of Sartana, including a list of members, was published on Facebook by the Minahasan journalist Rendai Ruauw (2019).

3 Ruauw (2019) includes some pictures of members and of their tattoos.

4
*Preman* often feature in movies, mostly in comic roles. Take, for example, the YouTube adventures of Sukri Basto, a *preman* in the city of Makassar for whom things always tend to go slightly wrong. He accidentally swallows a mouthful of chilies just when he must act really tough, the restaurant he decides to rob happens to be a police hang-out, etc.

5 The vast majority of *preman* are male, but there can occasionally be female *preman* (see Bakker and Karim 2022: 123–125).

6 See Barker (1998) and Siegel (1998, particularly pp. 90–120) for a more detailed discussion of Petrus.
